# A Novel Heterozygous Desmoplakin Variant Causes Cardiocutaneous Syndrome with Arrhythmogenic Cardiomyopathy and Palmoplantar Keratosis

**DOI:** 10.3390/jcm12030913

**Published:** 2023-01-24

**Authors:** Tolga Çimen, Argelia Medeiros-Domingo, Antonios Kolios, Deniz Akdiş, Shehab Anwer, Felix C. Tanner, Corinna Brunckhorst, Firat Duru, Ardan M. Saguner

**Affiliations:** 1 Department of Cardiology, University Heart Center, University Hospital Zurich, 8091 Zurich, Switzerland; 2 Swiss DNAlysis Laboratory, 8600 Dubendorf, Switzerland; 3 Department of Dermatology, University Hospital Zurich, 8091 Zurich, Switzerland; 4 Center for Integrative Human Physiology (ZIHP), University of Zurich, 8057 Zurich, Switzerland

**Keywords:** desmoplakin, arrhythmogenic cardiomyopathy, cardiocutaneous syndrome, repeat genetic testing

## Abstract

Cardiocutaneous syndrome (CCS) is often caused by genetic variants in desmoplakin (*DSP*) in the presence of thick calluses on the hands and soles of the feet (palmoplantar keratoderma) in combination with arrhythmogenic cardiomyopathy. In this case report, we describe a 58-year-old man presenting with a history of cardiomyopathy with recurrent sustained ventricular tachycardia and palmoplantar keratosis. The cardiological evaluation showed biventricular cardiomyopathy, and repeated genetic testing identified a novel *DSP* variant. Repeated genetic testingis clinically meaningful in patients with a high probability of a specific inherited cardiac disease, such as CCS, particularly if molecular screening has been performed in the pre-NGS era with an incomplete NGS panel or outdated technology as presented in this case report.

## 1. Introduction

Patients with desmoplakin (*DSP*) cardiomyopathy frequently have curly hair and/or thick skin on their palms or soles (palmoplantar keratoderma) and a left ventricular predominant arrhythmogenic cardiomyopathy (ACM) [1]. However, milder dermatologic manifestations in patients may have been unnoticed [2] and when assessing patients with biventricular cardiomyopathy in particular, physicians should be watchful for dermatological symptoms. Previously, it was shown that genetic retesting of patients with primary arrhythmia syndromes and cardiomyopathies, who initially tested negative for the core genes using traditional techniques, resulted in an additional 20% of genetic diagnoses with next-generation sequencing (NGS)-based extended gene panels [3]. In this case report, we discuss a 58-year-old man with palmoplantar keratosis and recurrent sustained ventricular tachycardia (VT) whose initial genetic analysis revealed no evidence of the prevalent ACM genes, but in whom a novel DSP variant was identified upon genetic retesting with state-of-the-art technology

## 2. Case Description

**Figure 1:**
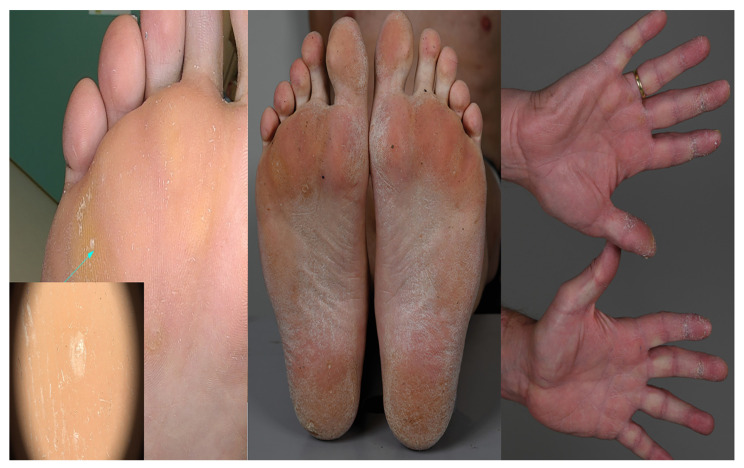
Symmetric palmoplantar keratoderma. Histological examination showed acanthosis with hypergranulosis and compact hyperkeratosis (arrow).

A 58-year-old man with a history of cardiomyopathy of unknown etiology was presented to our outpatient clinic for genetic counseling. His vital signs were unremarkable (heart rate 50 bpm, arterial blood pressure 103/84 mmHg). There were no signs of congestive heart disease, but a thorough physical examination revealed skin abnormalities located on the hands, feet, and knees (Figure 1), which were previously suspected to be psoriasis lesions.

**Figure 2:**
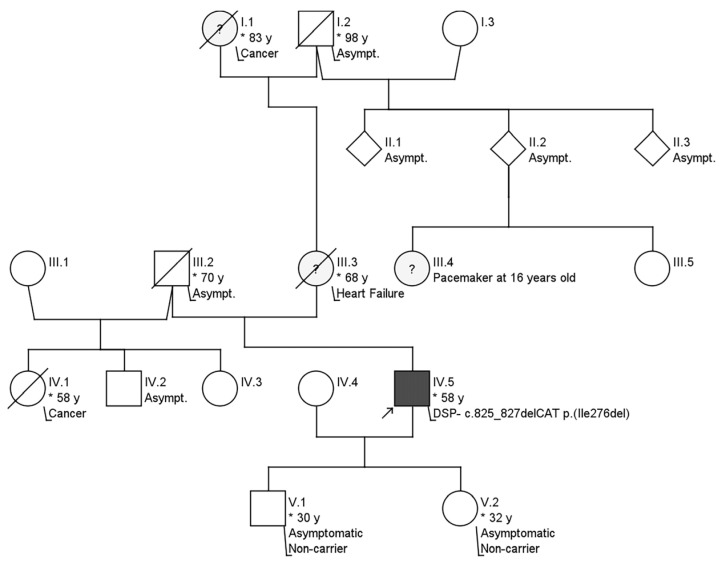
Pedigree of the family. No signs of further hereditary or contributing illnesses were present. IV.5 represents the index patient. * indicates the age in years.

The patient was a former athlete regularly engaging in endurance sports (rowing and running; 7 times/week) from puberty. When he was 35 years old, VT episodes appeared for the first time, which manifested themselves in several attacks of dizziness without fainting. At that time, the patient was suspected of having ACM, based on clinical and magnetic resonance imaging (MRI) findings. The patient’s family history regarding a hereditary heart disease was unremarkable over three generations (Figure 2). Secondary prophylactic implantable cardioverter-defibrillator implantation was carried out. After a stable clinical period, the patient was hospitalized for typical atrial flutter and heart failure at age 52. Ablation of typical atrial flutter improved left ventricular ejection (LVEF) from 15% to 27%. In order to confirm the diagnosis of suspected ACM, genetic testing using NGS was carried out in a referral center abroad using a panel that analyzes the genes desmocollin-2 (*DSC2*), desmoglein-2 (*DSG2*), desmoplakin (*DSP*), plakoglobin (*JUP*), lamin A/C (*LMNA*), plakophilin-2 (*PKP2*), transforming growth factor beta 3 (*TGFB3*), and transmembrane protein 43 (*TMEM43*) using Illumina MiSeq, 2 × 150 bp, paired end/HaloPlex Custom Kit (Agilent). No abnormal genetic variant was reported using this panel. He was again hospitalized due to an electrical storm, which was triggered by hypokalemia at age 53. He received catheter ablation of monomorphic sustained VT. Afterwards, new sustained VT episodes reoccurred, which were treated with amiodarone. The patient was stable on long-term amiodarone therapy.

## 3. Diagnostic Assessment

**Figure 3:**
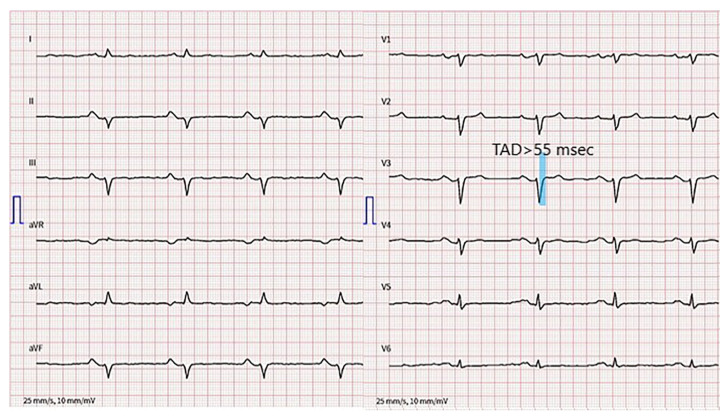
12-lead electrocardiogram. Terminal activation duration delay in V1–V3, left anterior hemiblock and PR prolongation are present.

Except for terminal activation duration delay (TAD) in V1–V3, the resting 12-lead ECG (Figure 3) does not meet the typical ACM nor arrhythmogenic right ventricular cardiomyopathy (ARVC) characteristics according to the 2010 ARVC Task Force (TFC) [4] and 2020 Padua criteria [5].

**Figure 4:**
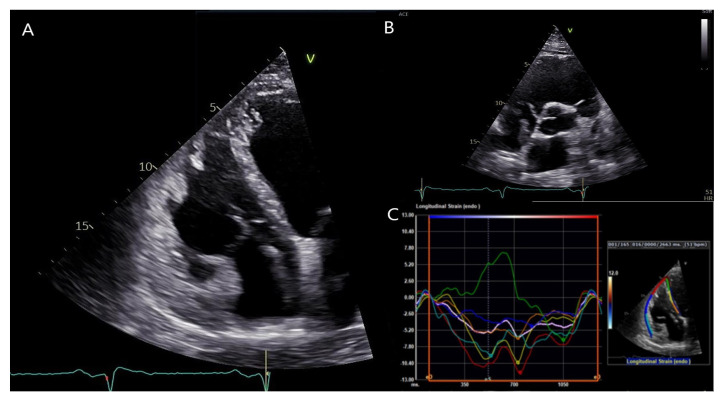
Transthoracic echocardiography of the patient. (**A**): Apical four-chamber view showing a dilated right ventricle (RV). (**B**): Parasternal short axis view showing RV outflow tract dilation. (**C**): RV free wall strain was significantly impaired (−7.7%). Segmental strain showed more impaired values for the septal, inferior and apical segments in both left ventricular and right ventricular segments, and mostly showing post-systolic shortening phenomenon. Usually, the septum is spared by the disease process in classical right dominant ARVC. Conversely, as illustrated by the green segmental strain curve, the RV apicoseptal region was dyskinetic in our patient with *DSP* cardiomyopathy.

Transthoracic echocardiography of the patient showed a dilated left ventricle (LV) with moderately reduced ejection fraction (EF), akinesia of the LV inferolateral segments together with hypokinesia of the remaining segments, severe RV dysfunction with dilatation of the RV/RVOT, and akinesia of the free wall. (Figure 4 and Video S1), fulfilling both major criteria from the 2010 TFC and 2020 Padua criteria [4,5]. Both left and right ventricular global longitudinal strain were notably impaired (−11.5 and −7.0%, respectively). Similarly, the RV free wall strain was impaired (−7.7%).

**Figure 5:**
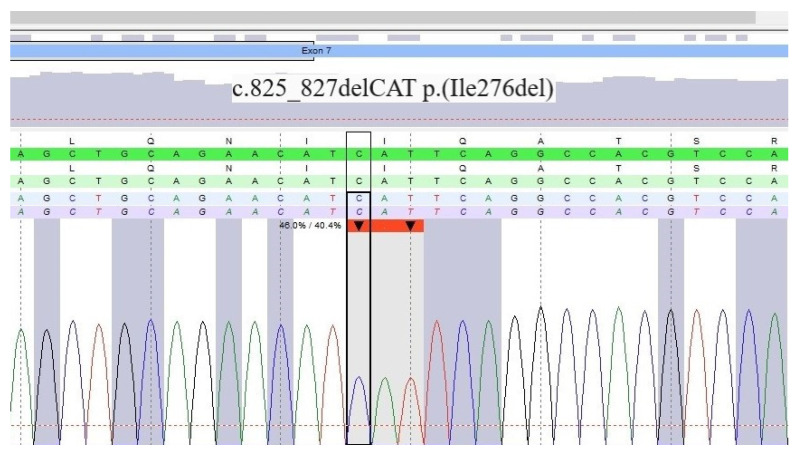
Result of DNA sequencing of the desmoplakin gene.

We performed positron emission tomography (18F-FDG PET/CT) and excluded sarcoidosis, a common differential diagnosis of ACM [6]. The patient’s skin lesions were also examined and cardiocutaneous syndrome (CCS) composed of ACM and palmoplantar keratosis was suspected. A skin sample taken from the plantar area indicated severe hyperkeratosis excluding psoriasis. Despite a previously negative genetic test and a negative family history, due to the high probability of CCS and incomplete NGS panel only including *PKP2*, *DSG2*, *DSP*, *DSC2*, *JUP*, *TGFB3*, *TMEM43* and *LMNA* performed abroad, we opted for repeat genetic testing using a custom cardiogenetic panel covering 173 genes (Agilent, Santa Clara, CA, USA, SureSelect^QXT^Target Enrichment) and NGS (Illumina MiSeq). We additionally used multiplex ligation-dependent probe amplification (MLPA, MRC Holland, Amsterdam, The Netherlands) for *DSC2*, *DSG2*, *DSP*, *JUP*, *PKP2*, phospholamban (*PLN*), *TGFB3*, and ryanodine receptor 2 (*RYR2*). In the second genetic test, we identified a novel heterozygous single amino acid deletion variant localized in the domain in exon 7 of the *DSP* gene that interacts with plakoglobin (Figure 5), which was confirmed by Sanger sequencing. This deletion variant *DSP* (NM_004415.4):c.825_827del (p.Ile276del) has not yet been described in the literature and is currently classified by the American College of Medical Genetics criteria as a variant of unknown significance (class III).

The identification of a new heterozygous variant in the *DSP* gene fitted well with the diagnosis of ACM with biventricular involvement and palmoplantar keratosis. Therefore, suspicion for the pathogenicity of the new variant is high and we suggest an upgrade to the pathogenic classes IV or V.

The patient received cardiogenetic counseling, including a recommendation for cascade screening of his first-degree family members. Genetic testing of his two phenotypically negative children for this *DSP* variant was negative. Moreover, the index patient was advised to decrease the intensity of his sports activity.

## 4. Discussion

A heterozygous variant in *DSP* in a single family has been previously identified following the publication of the causes of CCSs Naxos disease and Carvajal syndrome [7]. These researchers eventually discovered other families with ACM and other dominant *DSP* variants, observing that LV involvement was common in these families [8]. *DSP* cardiomyopathy affects the LV in many cases, with or without RV involvement [1], which stands in contrast to *PKP2* cardiomyopathy, which predominantly affects the RV. Our patient was an athletic patient and experienced severe ventricular arrhythmic episodes coexisting with overt LV systolic dysfunction. In addition to these clinical clues, skin lesions were suspicious for *DSP* cardiomyopathy and altogether enabled us to establish the correct diagnosis. As recently observed for autosomal dominant *DSP* variants, the presence of curly hair and/or thick-callused skin on the palms and/or soles of the feet (palmoplantar keratoderma) increases the likelihood to diagnose *DSP* cardiomyopathy [9]. Despite the fact that de novo variants in ARVC have been found on occasion, they only account for 1.4% of all desmosomal variants [10]. Because the patient’s family history suggested no hereditary conditions, we were unable to determine whether or not this was a familial or sporadic type due to insufficient genetic testing. The patient’s history of endurance exercise may be the most significant characteristic separating him from other family members, enhancing phenotypic penetrance and disease progression [11].

Repeat genetic testing is feasible if test sensitivity has increased or if the patient’s phenotype is compatible with a newly found gene. We performed further inquiry to the laboratory in which the first genetic test was carried out in 2016, to assess the reason of missing the *DSP-* c.825_827delCAT p.(Ile276del) variant. The conclusion was that the variant was filtered out by the bioinformatics pipeline (SeqNext by JSI medical systems and software developed by that center) setup back then. The latest expert consensus statement on the state of genetic testing for cardiac diseases strongly recommends repeat genetic testing in patients with a high probability of a specific inherited cardiac disease and if molecular screening has been performed in the pre-NGS era or with an incomplete NGS panel [12]. In a recent study, it was shown that repeat genetic testing in primary arrhythmia syndromes and cardiomyopathy resulted in an additional genetic diagnosis in up to 20% of the cases [3]. Despite the first genetic testing being carried out in a referral tertiary care center, repeat genetic testing was worthwhile because the phenotype fitted well with *DSP* cardiomyopathy/CCS.

## 5. Conclusions

We describe a case with biventricular ACM and palmoplantar keratosis. The patient tested previously negative for common ACM genes including *DSP*, but repeat genetic testing with state-of-the-art technology identified a new *DSP* variant. It can be worthwhile to consider updating the old genetic test in patients whose clinical findings strongly suggest *DSP*-cardiomyopathy with CCS.

## Data Availability

Upon urgent request and associated need, our data are available, while our upmost intention is to protect our patients’ privacy.

## Supplementary Materials

The following supporting information can be downloaded at: https://www.mdpi.com/article/10.3390/jcm12030913/s1, Video S1: Apical four-chamber view showing a dilated right ventricle (RV) with severe dysfunction, akinesia of the RV free wall and paradoxical movement of the apical septum.
